# Characterizing tuberculosis transmission dynamics in high-burden urban and rural settings

**DOI:** 10.1038/s41598-022-10488-2

**Published:** 2022-04-26

**Authors:** Jonathan P. Smith, John E. Oeltmann, Andrew N. Hill, James L. Tobias, Rosanna Boyd, Eleanor S. Click, Alyssa Finlay, Chawangwa Mondongo, Nicola M. Zetola, Patrick K. Moonan

**Affiliations:** 1 Department of Health Policy and Management, Yale School of Public Health, 60 College Street, New Haven, CT 06510 USA; 2 Peraton, 2800 Century Pkwy NE, Atlanta, GA USA; 3 Division of Global HIV and Tuberculosis, Centers for Disease Control and Prevention, Atlanta, GA USA; 4 Division of Tuberculosis Elimination, Centers for Disease Control and Prevention, Atlanta, GA USA; 5 Botswana-UPenn Partnership, University of Pennsylvania, Philadelphia, USA

**Keywords:** Tuberculosis, Statistical methods

## Abstract

*Mycobacterium tuberculosis* transmission dynamics in high-burden settings are poorly understood. Growing evidence suggests transmission may be characterized by extensive individual heterogeneity in secondary cases (i.e., superspreading), yet the degree and influence of such heterogeneity is largely unknown and unmeasured in high burden-settings. We conducted a prospective, population-based molecular epidemiology study of TB transmission in both an urban and rural setting of Botswana, one of the highest TB burden countries in the world. We used these empirical data to fit two mathematical models (urban and rural) that jointly quantified both the effective reproductive number, $R$, and the propensity for superspreading in each population. We found both urban and rural populations were characterized by a high degree of individual heterogeneity, however such heterogeneity disproportionately impacted the rural population: 99% of secondary transmission was attributed to only 19% of infectious cases in the rural population compared to 60% in the urban population and the median number of incident cases until the first outbreak of 30 cases was only 32 for the rural model compared to 791 in the urban model. These findings suggest individual heterogeneity plays a critical role shaping local TB epidemiology within subpopulations.

## Introduction

Tuberculosis (TB), an airborne infectious disease caused by *Mycobacterium tuberculosis*, is a major global epidemic with an estimated 10 million new cases and 1.5 million deaths annually^1^. TB incidence varies widely from country to country, ranging from less than 10 cases per 100,000 people in almost all high-income countries, to over 500 per 100,000 people in several low- and middle-income countries^1^. These remarkable disparities suggest substantial reductions in incidence are achievable if policymakers are able to identify population-specific factors that determine TB transmission and implement targeted preventive measures^2–5^.

Understanding TB transmission dynamics is a notorious challenge given the marked variation in timing between infection and clinical TB disease. Limited evidence suggests TB transmission may be characterized by a high degree of individual heterogeneity, or differences in the number of secondary cases caused by each infectious individual^6–10^. Such heterogeneity implies outbreaks are rarer but more extensive, and has profound implications in infectious disease control^11–14^. However, such research has overwhelmingly focused on individual outbreaks in low-burden settings, and the degree and population-level influence of individual heterogeneity in high-burden TB populations remains unknown^14,15^. Researchers have explicitly called for an improved understanding of individual heterogeneity in high-burden populations^3,6,14–17^.

Accurately identifying discrete transmission events is further complicated in high-burden TB settings due to the considerable prevalence in the population; individual-level transmission events are almost always unobserved^18^. This precludes our ability to reconstruct accurate chains of transmission (e.g., transmission trees) required to assess individual heterogeneity in secondary cases ^19^. However, molecular characterization of *M. tuberculosis* isolates combined with other epidemiologic (i.e., geospatial, social contact, etc.) data has been shown to reasonably approximate entire TB transmission cluster sizes in high-burden settings, defined as the simple sum of all cases in a chain of recent transmission^6,7,18,20^. Since transmission chains give rise to transmission clusters, there exists an intrinsic relation between the distribution of individual secondary cases and entire cluster distributions^21–23^. Epidemiologic models have long exploited this relationship and have used the distribution of cluster sizes to infer the average number of secondary cases per each individual (known generally as the reproduction number, $R$) in other infectious diseases^21–25^. Extending the use of transmission cluster distributions to the problem of quantifying individual heterogeneity in TB transmission may provide more complete insight into transmission dynamics in in high-burden settings^10^.

Botswana has one of the highest TB incidence rates in the world and recognized by the World Health Organization as one of the top 30 high TB/HIV countries^1^. In this study, we prospectively collected detailed clinical, epidemiologic, geospatial, and genotypic data to characterize TB cluster distributions in both urban and rural settings of Botswana. We used these data to identify detailed TB genotypic and transmission subclusters through geospatial and social network analysis. Following previous studies, we used the distribution of TB transmission clusters to infer both the reproductive number $R$ and the propensity for superspreading by virtue of the negative binomial dispersion parameter $k$^7,26,27^. The dispersion parameter $k$ quantifies overdispersion in the distribution, with lower values of $k$ (i.e., $k \ll 1$) implying a greater propensity for superspreading^19^.

We used empirical estimates of transmission and individual heterogeneity to develop individual-based models for each setting (urban and rural). We compared underlying transmission dynamics between settings by testing scenarios where model parameters were unique, partially similar, or identical. We used the best fitting model in each setting to examine the population-level impact of individual heterogeneity by calculating (1) the proportion of secondary cases attributable to infectious cases, (2) the probability of a large outbreak (i.e., superspreading event), and (3) the number of incident cases until the first large outbreak. We also evaluated the degree and direction of potential bias in empirical estimates by conducting a full simulation study modeling common limitations in TB observation, including missing cases, censorship, and imperfectly defined transmission clusters.

## Methods

### Sources of data

We conducted a prospective, population-based molecular epidemiologic study aimed at identifying TB transmission networks within Botswana (the *Kopanyo* study, *“people gathering together”* in local Setswana language)^28,29^. Study participants were recruited from two geographically distinct districts of Botswana: the greater Gaborone district, which contains the City of Gaborone and its surrounding suburbs, and the rural Ghanzi district. Gaborone is the political and economic capital and the most populated urban center of Botswana, with a population density of approximately 1400 people per square kilometer and a TB notification rate of approximately 440–470 cases per 100,000 population^29^. Ghanzi is a large, rural agricultural district in northwest Botswana with an estimated population density of 0.4 people per square kilometer and an estimated TB notification rate of 722 per 100,000 population^29^. The HIV prevalence in the general population in both regions at the start of the study timeframe was an estimated 17 percent^30^.

All patients with a clinical or culture-confirmed TB diagnosis between 2012 and 2016 in Botswana’s greater Gaborone and Ghanzi districts were eligible for the study. Participants were recruited from all TB clinics and directly observed treatment centers in greater Gaborone (n = 24) and Ghanzi (n = 6). Only patients on treatment for 14 days or more prior to screening, incarcerated patients, or those refusing to consent were excluded from the study; additional details are available in the Supplementary Materials and a full protocol is available elsewhere^28^. Social, behavioral, clinical, and demographic data were obtained by both medical record abstraction and standardized patient interview. Geospatial data were obtained and validated by geotagging the latitude and longitude of each participant’s primary residence, place of work, and locations of social gathering venues (i.e., places of worship, alcohol-related venues, etc.). Culture isolates were genotyped using a standardized protocol for 24-locus mycobacterial interspersed repetitive units—variable number of tandem repeats (MIRU-VNTR)^31^. Participants with matching MIRU-VNTR results were considered in the same genotypic cluster.

In December 2020, we retrospectively abstracted clinical encounter data from the Botswana Ministry of Health’s universal electronic medical record (EMR) database known as the Integrated Patient Management System (IPMS). IPMS contains detailed information on all clinical encounters in Botswana regardless of indication and it has been deployed to the vast majority of public and private clinics since the late 2000s (See supplemental materials Sect. 1.9). We abstracted all clinical encounters for Kopanyo participants in the IMPS database occurring between March 1, 2004 and December 31, 2018.

### Transmission sub-cluster definition

**Figure 1:**
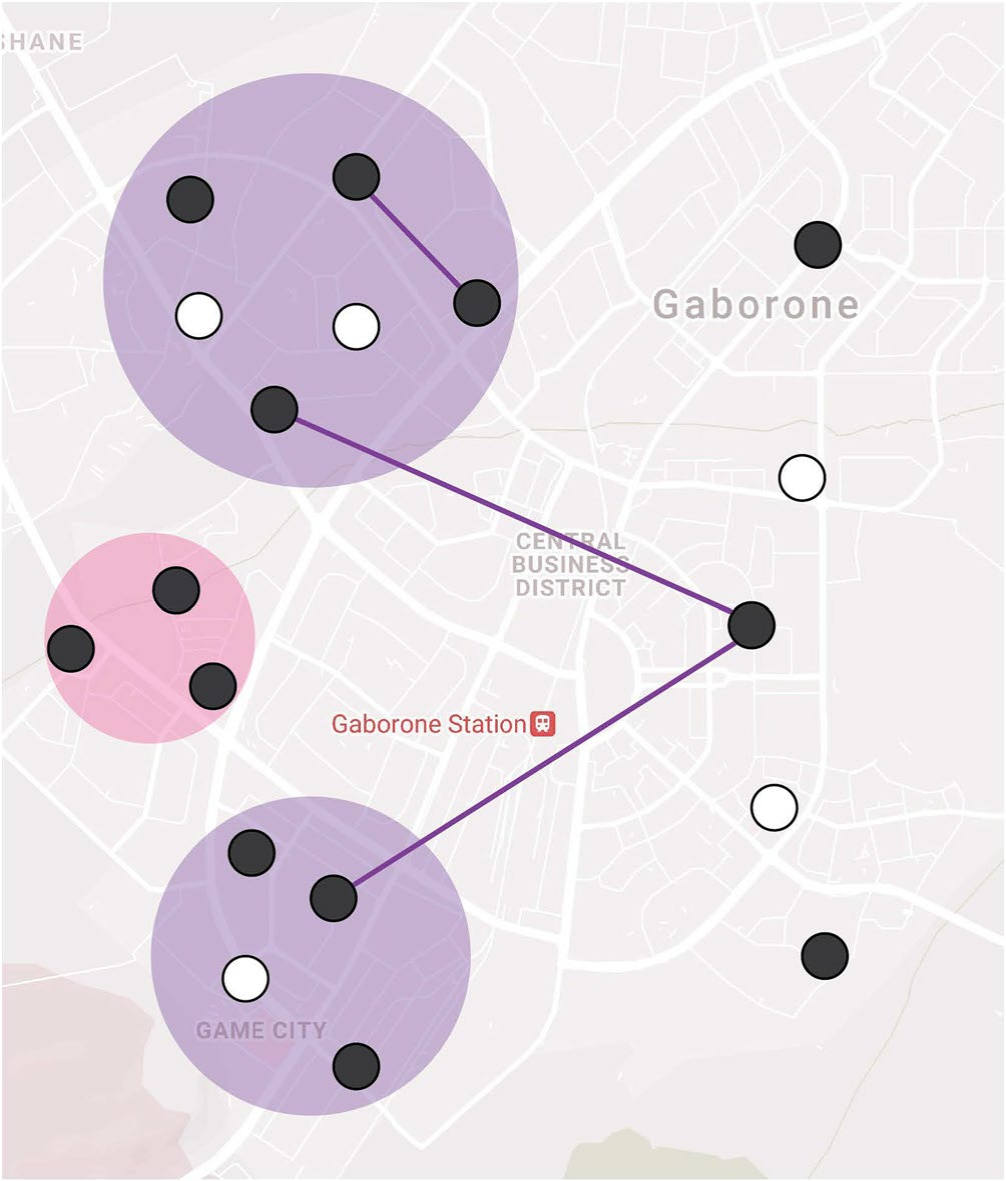
Visualization of primary transmission sub-cluster definition. Using geospatial and epidemiological data, we estimated the size of TB transmission sub-clusters within a MIRU-VNTR genotypic cluster. Participants with the same *M.* tuberculosis genotype (black circles) were considered a genotypic cluster ($Y = 13$). Participants were in a transmission sub-cluster if they were in the same SaTScan geospatial cluster (colored circles) or identified through an epidemiological link (solid lines). This fictitious example represents a genotypic cluster of size 13 with two transmission sub-clusters of size 8 (purple) and 3 (pink) and two isolated cases. White circles represent TB cases with a different MIRU-VNTR profile and are not included in the cluster definitions.

MIRU-VNTR-based clusters may contain multiple transmission sub-clusters and overestimate TB transmission^6,7,32^. We defined transmission sub-clusters as patients with the same genotype results identified in a geospatial cluster or having an epidemiologic link (Fig. 1). Geospatial clusters were defined using SaTScan (https://www.satscan.org; v9; see supplemental materials Sect. 1.3)^33^. Epidemiologic links (epi-links) were defined as patients with the same genotype results that frequented the same (i.e., place of work, place of worship, alcohol-related venue, etc.), resided at the same address, or had an overlapping clinical encounter during the putative infectious period (supplemental materials Sect. 1.6). To relate epi-links more plausibly to recent transmission, only patients with one or more epi-links and enrolled within two years were considered a possible transmission link, with exceptions related to healthcare facilities (supplemental materials Sect. 1.6). Cases with no established geospatial or epidemiologic link were considered isolated cases. Transmission clusters containing at least five participants with an incident case diagnosed within two years of the end of the study timeframe were considered censored (e.g., ongoing at the time of data collection).

### Modeling framework

We developed two primary models, an urban and rural model, based on if the participant’s primary residence was in the greater Gaborone district or Ghanzi district, respectively. Both models were based on a classical Galton-Watson branching process^10,21,23^. Briefly, each individual case in the population was assigned an associated individual reproductive number, denoted $\nu$, drawn from a given probability distribution with mean $R$^11^. $\nu$ represents the expected number of secondary TB cases for each individual; the observed number of cases, denoted $Z$, is a consequence of $\nu$ and demographic stochasticity. Following previous studies, we assumed $\nu$ is gamma distributed with mean $R$ and dispersion parameter $k$ and modeled demographic stochasticity using a Poisson process^11,21,34^. This Poisson-gamma mixture yields a negative binomial distribution of secondary TB cases (offspring distribution), also with mean $R$ and dispersion $k$. The free parameter $k$ quantifies the degree of individual heterogeneity and is a measure of overdispersion in the distribution. Lower $k$ values indicate increased individual heterogeneity, with values of $k < 1$ representing a high propensity for extensive recent transmission. Detailed methods are available in Sect. 1.1 of the supplemental materials and the full code is available at https://github.com/jpsmithuga/UrbanRural_nbbpAnalysis.

### Parameter inference from cluster data

We used maximum likelihood estimation (MLE) to jointly infer negative binomial model parameters $R$ and $k$ from empirical transmission cluster size distributions (see supplemental materials Sect. 1.1.1–1.1.5)^22,23,27,35^. We made mechanistic adjustments to account for two limitations common in cluster sizes, denoted $Y$. First, exact transmission sub-clusters may not always be cleanly and unambiguously identified within the larger genetic cluster. Disentangling transmission sub-clusters from larger genotypic clusters ad hoc has been shown to introduce bias^27^. Instead, we conditioned the probability of a genotypic cluster reaching final size $Y$ on the number of transmission subclusters, $n$, identified in the genotypic cluster (i.e., $P\left( {Y = y{|}n} \right)$; See supplemental materials Sect. 1.1.4)^22^. This approach considers all possible ways $n$ transmission subclusters can result in a final genetic cluster size of $Y$. Second, we considered clusters that are ongoing at the time of data collection to be censored. We accounted for censoring by considering the probability that a censored genetic cluster of size $Y$ containing $n$ transmission subclusters was at least size $Y$ (i.e., $P\left( {Y \ge y{|}n} \right)$; See supplemental materials Sect. 1.1.6)^7,23^. Confidence intervals (95% CI) were obtained through profile likelihood^36^. Using inferred parameters we also calculated the expected proportion of cases responsible for all secondary transmission (Supplemental Materials Sect. 1.5).

### Comparing transmission dynamics between settings

Using model likelihoods we determined if there was statistical support for differences in transmission dynamics between the urban and rural populations^37^. Let $R_{u} , k_{u}$ and $R_{r} , k_{r}$ represent the parameters for the urban and rural models, respectively. We developed six comparison models by placing restrictions on model parameters: (1) an unrestricted model, with no restrictions on model parameter $s;$ (2) an identical transmissibilities model, which restricts $R$ values to be the same ($R_{u} = R_{R}$); (3) an identical heterogeneities model, which restricts $k$ values to be the same, ($k_{u} = k_{r}$); (4) a fully identical model, which forces all parameters to be the same for both populations ($R_{u} = R_{r}$ and $k_{u} = k_{r}$); (5) an SIR-type heterogeneity model which assumes $k_{u} = k_{r} = 1$ and; (6) an SIR-type identical model, which assumes $R_{u} = R_{r}$ and $k_{u} = k_{r} = 1$. The latter two SIR-type models correspond to common assumptions made in typical differential-equation models of homogenous mixing and constant infectivity over an exponentially distributed infectious period^38–40^. We compare model fit using the Akaike Information Criterion (AIC); the lowest AIC score determined the best fitting model. We then calculated information loss, which provides a measure of how likely the best fitting model explains the observed data relative to the comparison models^41,42^.

### Sensitivity analysis and model evaluation

We evaluated three primary sources of potential bias: the appropriateness of model assumptions, the definition of transmission clusters, and the sensitivity of parameter inference. Briefly, we compared models with two alternative distributional assumptions of $\nu$ (constant and exponential). We also considered three alternative transmission sub-cluster definitions, including a MIRU-only definition that inappropriately assumes genotypic clusters were themselves wholly observed transmission clusters. This assumption biases transmission towards homogeneity, and thus provides a functional upper bound estimate of heterogeneity in the models. Lastly, we conducted a detailed simulation study to assess the sensitivity of parameter inference to data limitations. We simulated perfect and imperfect surveillance systems under $R$ and $k$ parameters inferred from the urban and rural cluster distributions. Perfect surveillance was defined as all cases completely observed with no censoring or sub-clustering. Imperfect surveillance was subject to biases imposed by (1) incomplete case observation (i.e., the probability that a case is identified, produces a culture-positive result, and yields interpretable genotypic results), (2) active case finding (i.e., an otherwise unobserved case identified by contact tracing), (3) sub-clustering, or the inability to unambiguously tease apart multiple transmission clusters, and (4) censoring, or ongoing clusters at the time of data collection (Supplemental Figure S1). Detailed methods and results of all sensitivity analyses can be found in the supplementary materials, Sects. 1.2–1.5.

### Ethics

This study was approved by the Centers for Disease Control and Prevention (CDC) Institutional Review Board (IRB); the Health Research and Development Committee, Ministry of Health and Wellness, Botswana; and the University of Pennsylvania IRBs. All methods were carried out in accordance with relevant guidelines and regulations. Informed consent was obtained from all subjects and/or their legal guardian(s).

## Results

### Cluster data and parameter inference

A total of 4331 cases were enrolled in the study, of which 3736 (86%) had a validated geocoded primary residence, 3891 (90%) had EMR data linked from the IPMS database, and 2137 (49%) had culture-positive, pulmonary tuberculosis with genotyping data available; 1683 (39%) had combined genotypic, geospatial, and epidemiologic data suitable for analysis (Table 1). Among these, 1290 (77%) were included in the urban population and 393 (23%) were included in the rural population (Table 1). There were no statistically significant differences in culture status, age, gender, successful assignment of MIRU-VNTR profile, obtaining EMR data, or validated geocoded address between patients included and not included in the analysis. The urban population contained 564 distinct MIRU-VNTR clusters; 386 urban participants (30%) were isolated cases. The rural population contained 114 distinct MIRU-VNTR clusters with 73 (19%) isolated cases. The largest genotypic cluster in the urban population was a censored cluster of size 78 with 15 transmission sub-clusters; in the rural population the largest cluster was a censored cluster of size 147 with 25 transmission sub-clusters.

**Table 1:** Genotypic cluster and case distributions, by population.

Genotypic cluster size	Urban		Rural	
	Clusters, n (%)	Cases, n (%)	Clusters, n (%)	Cases, n (%)
1	386 (68)	386 (30)	73 (64)	73 (19)
2	75 (13)	150 (12)	19 (17)	38 (10)
3	39 (7)	117 (9)	5 (4)	15 (4)
4	17 (3)	68 (5)	7 (6)	28 (7)
5	5 (1)	25 (2)	1 (1)	5 (1)
6	7 (1)	42 (3)	2 (2)	12 (3)
7	7 (1)	49 (4)	0 (0)	0 (0)
8	4 (1)	32 (2)	2 (2)	16 (4)
9	5 (1)	45 (3)	1 (1)	9 (2)
≥ 10	19 (3)	376 (29)	4 (4)	197 (50)
Total	564 (100)	1290 (100)	114 (100)	393 (100)

**Figure 2:**
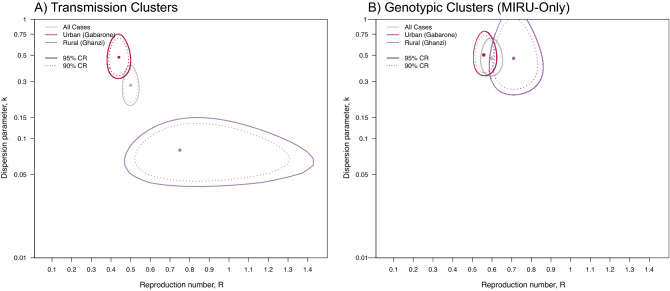
Joint maximum likelihood estimates (MLE) of transmission parameters $R$ and $k$ inferred from transmission cluster analysis. The use of geospatial and epi-link data to identify transmission sub-clustering reveals marked differences in transmission dynamics. (**A**) MLE and corresponding 90 and 95 percent confidence regions (CRs) using genotypic, geospatial, and epi-link data to identify transmission sub-clusters within a genetic cluster; (**B**) Values under the assumption that MIRU-VNTR genotypic clusters are transmission clusters. This assumption biases results towards homogeneity and provides functional upper bound estimates of $k$.

The maximum likelihood estimates (MLE) of $R$ were 0.44 (95% CI: 0.39–0.50) in the urban population and 0.75 (95% CI: 0.48–1.46) in the rural population (Table 2; Fig. 2, Panel A); MLE estimates of $k$ were 0.48 (95% CI: 0.31–0.87) and 0.08 (95% CI: 0.04–0.14) in the urban and rural populations, respectively.

**Table 2:** Maximum likelihood estimates (MLE) for $R$ and $k$ by population. The primary analysis used epidemiologic, geospatial, and genotypic data to define transmission subclusters. The sensitivity analysis assumed MIRU-VNTR genotypic clusters were transmission clusters.

	Primary analysis		Sensitivity analysis (“MIRU-only”)	
	$\hat{R}$(95% CI)	$\hat{k}$(95% CI)	$\hat{R}$(95% CI)	$\hat{k}$(95% CI)
Urban	0.44 (0.39–0.50)	0.48 (0.31–0.87)	0.56 (0.51–0.62)	0.50 (0.34–0.79)
Rural	0.75 (0.48–1.46)	0.08 (0.04–0.14)	0.71 (0.59–0.86)	0.47 (0.24–1.10)

### Comparison of transmission dynamics between urban and rural populations

**Figure 3:**
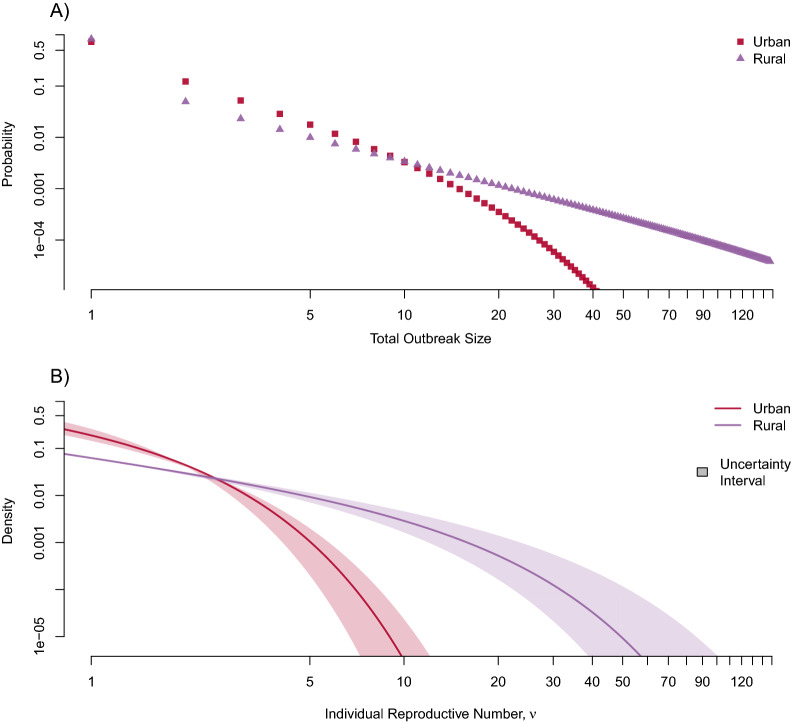
Underlying transmission dynamics in urban and rural models. We fit urban and rural models to the distribution of transmission cluster size data to infer the degree of individual heterogeneity in secondary cases. While all models show that TB transmission is characterized by a high degree of individual heterogeneity, the rural model suggests a substantially higher propensity for explosive outbreaks of recent transmission. (**A**) Probability of observing large outbreaks originating from a single index case; (**B**) Probability density of expected number of secondary cases for each individual (i.e., underlying individual reproductive number, $\nu$). The uncertainty interval integrates across the entire range of 95% confidence intervals for both $\hat{R}$ and $\hat{k}$**.**

**Figure 4:**
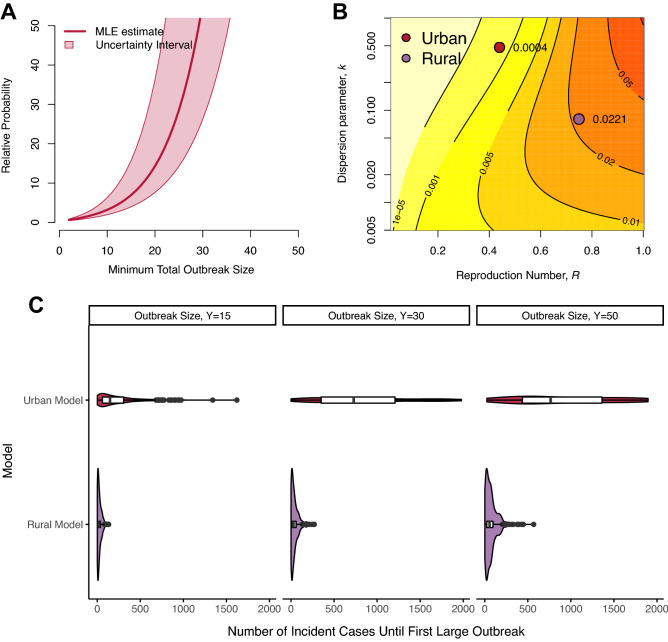
Comparison of large TB outbreaks in high-burden urban and rural settings. (**A**) Relative probability of observing a large outbreak of at least size $Y$ generating from a single case in a rural population compared to an urban population; (**B**) Absolute probability that a single case results in an outbreak of size of 30 or greater. Colored contours indicate probability bands, with associated probabilities indicated on each band. Setting-specific estimates are provided for clarity. (**C**) Density curves for the number of incident cases until first observed outbreak of size $Y = 15$, $Y = 30$, and $Y = 50$ resulting from a single index case. Nested boxplots represent the median and interquartile range of 500 simulated surveillance systems, each with 2000 transmission chains (supplemental materials Sect. 1.8). All $Y$ values were arbitrarily chosen to represent sufficiently large outbreaks.

The two populations had markedly different underlying mechanisms of TB transmission (Fig. 3). The rural population was substantially more likely to experience larger transmission events relative to the urban population (Fig. 4). For instance, the rural model was 64 (95% CI: 30–196) times more likely to observe an outbreak of size 30 compared to the urban model, and the median number of incident cases until the first outbreak of size 30 was only 31.5 (IQR: 13–60) for the rural model compared to 791 (IQR: 402–1253) in the urban model.

Of the six models developed to compare transmission dynamics in the two populations, the unrestricted model, which assumed the populations had entirely unique transmission parameters, best supported the observed data (Table 3). The identical transmissibility model, which assumed both populations had the same $R$ value, was the second-best fitting model yet was four times less likely to explain the observed data than the unrestricted model. All other models, including the assumption of interest that the two populations had identical heterogeneity, were at least four orders of magnitude less likely to explain the data (< 1/10,000).

**Table 3:** Model results for comparing transmission dynamics in the urban and rural populations under various model assumptions.

Model	Model assumptions/description	Model parameter restrictions	∆ likelihood	AIC	∆ AIC	Relative information loss
Unrestricted	Populations have different transmission dynamics	None	*Reference*	1766.67	*Reference*	*Reference*
Identical transmissibility	Populations have same transmission potential but different heterogeneities	$R_{u} = R_{r}$	− 2.43	1769.52	− 2.85	~ 1/4
Identical heterogeneity	Populations have different transmission potential but same heterogeneity	$k_{u} = k_{r}$	− 12.39	1789.45	− 22.78	~ 1/88,470
Fully identical	Populations have identical transmission dynamics	$R_{u} = R_{r}$ $k_{u} = k_{r}$	− 14.05	2543.41	− 24.10	~ 1/3,723,000
SIR-type heterogeneity	Populations have different transmission potential and SIR-type heterogeneity	$k_{u} = k_{r} = 1$	− 12.50	1787.67	− 21.10	~ 1/36,406
SIR-type identical	Populations have same transmission potential and SIR-type heterogeneity	$R_{u} = R_{r}$ $k_{u} = k_{r} = 1$	− 17.13	1794.93	− 28.27	~ 1/1,373,130

**Figure 5:**
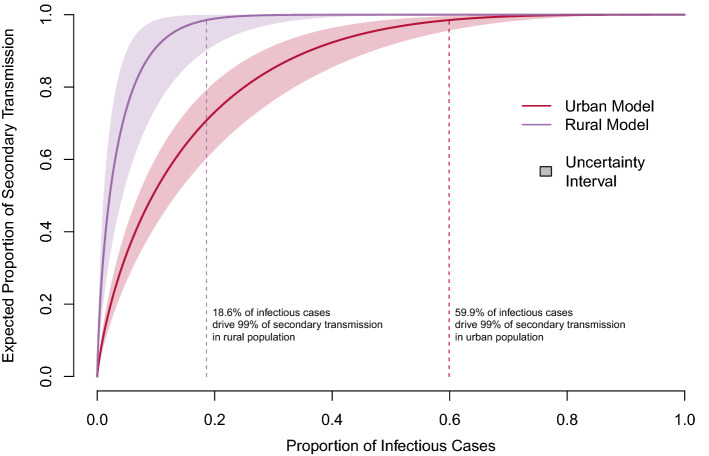
Expected proportion of TB transmission attributed to a given proportion of infectious cases, by population. The proportion of cases responsible for 99 percent of transmission in each model is denoted by the vertical dotted line. The uncertainty interval integrates across the entire range of 95% confidence intervals for both $\hat{R}$ and $\hat{k}$.

Both models estimated that most secondary transmission was attributable to a minority of infectious cases, yet the proportion of cases responsible for secondary transmission varied considerably between models (Fig. 5). For instance, an estimated 80 percent of secondary cases were attributable to 25 percent of infectious cases in the urban model, yet that proportion was four times less in the rural model (6 percent). Similarly, around 60 percent of infectious cases were responsible for 99 percent of secondary cases in the urban model, compared to only 20 percent in the rural model (Fig. 5).

### Sensitivity analysis

Our assumption of a gamma-distributed $\nu$ was superior in fitting the observed data compared to the exponential or constant $\nu$ assumption for all models (Supplementary Figures S2 and S3). Our alternative transmission cluster definitions resulted in expected under- and overestimation of heterogeneity (Supplemental Figure S4). The MIRU-only definition provides the most conservative estimates of heterogeneity and biased $\hat{k}$ towards homogeneity (Fig. 2B, Supplementary Figure S4), yet still implied a high degree of heterogeneity in both populations( $\hat{k} = 0.50$ (95% CI: 0.37–0.79) and $\hat{k} = 0.47$ (95% CI: 0.24–1.10) for the urban and rural populations, respectively).

**Figure 6:**
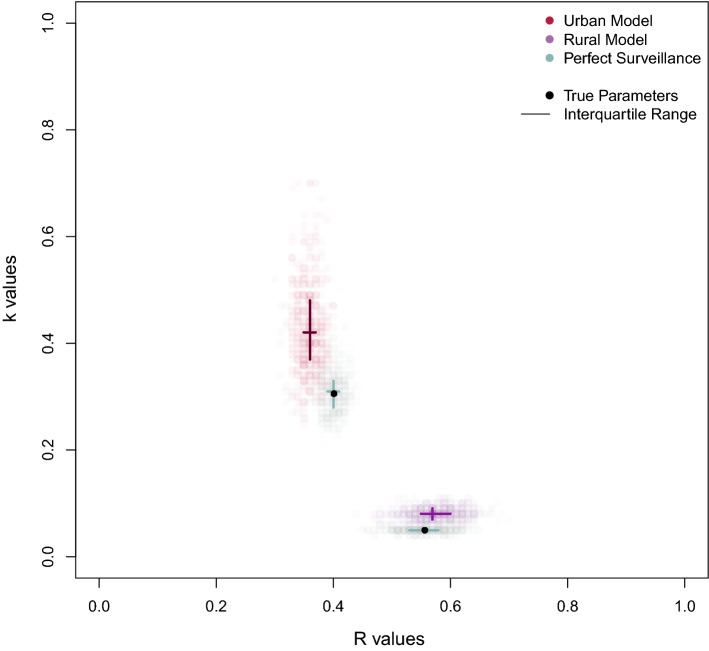
Sensitivity of model inference to imperfect surveillance. We simulated 500 perfect and imperfect TB surveillance systems for both the urban and rural models, each with 2000 chains of transmission. True underlying $R$ and $k$ values were specified by the inferred values from the respective populations. In perfect surveillance, all cases were observed with no censoring or sub-clustering. Imperfect simulations combined the following assumptions: only 40% of cases were observed ($p_{1} = 0.40$) and only 15% of otherwise missing cases were identified by active case finding ($p_{2} = 0.15$) for both models. The proportion of censored clusters ($p_{cens}$) and genotypic clusters containing multiple sub-clusters ($p_{over}$) were consistent with the observed values ($p_{cens} = 0.07$ and $0.07$ and $p_{over} = 0.15$ and $0.28$ for the urban and rural models, respectively). See supplemental materials for detailed methods and results from the simulation study.

Our cluster-based inference performed accurately and equally well under perfect surveillance conditions when compared to standard approaches using known individual-level data (i.e., transmission trees), with a median $\hat{k} = 0.48$ vs true $k = 0.48$ in the simulated urban population and median $\hat{k} = 0.08$ vs true $k = 0.08$ in the simulated rural population (Supplemental Figure S5, Fig. 6). Under assumptions that only 40% of cases were observed, only 15% of missed cases were later obtained through active case finding, and censoring and sub-clustering were consistent with observed values, inference of $\hat{k}$ was slightly biased upwards (towards homogeneity) in both models (median $\hat{k} = 0.63$ vs true $k = 0.48$ and median $\hat{k} = 0.14$ vs true $k = 0.08$ in the simulated urban and rural populations, respectively; Fig. 6), suggesting transmission in both populations may be more heterogeneous than estimated. Importantly the models could clearly distinguish between the two populations despite these introduced biases (Fig. 5).

## Discussion

This analysis revealed remarkable differences in TB transmission dynamics between urban and rural populations in a high-burden TB and HIV setting. While we emphasize that both populations were characterized by extensive outbreaks of recent transmission ($k < 1$), the rural population demonstrated a substantially higher propensity for such events. These findings have important implications for TB policy programs seeking to interrupt transmission and suggest that early identification of TB clusters may have a disproportionate impact in further reducing TB incidence in the rural population compared to the urban population. Our results also establish empirical estimates quantifying heterogeneity in high-burden settings, allowing for future work to evaluate the impact of specific intervention strategies to represent the mechanisms underlying TB transmission more accurately.

Our findings are in stark contrast to the common perception in infectious diseases that densely populated urban areas are more prone to explosive outbreaks than sparely populated rural areas. These findings likely do not reflect individual-level factors but instead can be attributed to several underlying environmental, social, and cultural mechanisms. Urban dwellers may live in closer proximity to healthcare facilities and more readily access antituberculosis treatment and care relative to rural dwellers, thus reducing the duration of infectiousness. In addition, rural populations may have fewer venues for social congregation (e.g., churches, alcohol-related venues, etc.). Fewer venues might concentrate a larger proportion of the population in these venues and result in a higher proportion of transmission relative to urban venues. Differences in social practices between populations may also provide insight; inhabitants of Ghanzi, like many populations in rural southern Africa, experience seasonal oscillating migration between their residence and towns and villages for employment and other economic and cultural reasons, often residing in temporary congregate lodging ^29^. Such migration has been shown to increase the risk of both acquiring and transmitting TB in similar populations^43–45^.

We performed multiple sensitivity analyses to evaluate model development and inference. We first compared multiple underlying assumptions of heterogeneity in the model, the evaluated differences between the populations using six comparison models. We also undertook a comprehensive simulation study both to verify that cluster-size distributions were sufficient for parameter inference and to evaluate the impact that imperfect surveillance has on the inference procedure. All analyses supported the primary findings that transmission in both populations was characterized by a high propensity for extensive transmission, and that rural populations had a higher probability of experiencing large outbreaks compared to the urban population. Importantly, despite a high degree of bias introduced, the models were able to easily distinguish between the urban and rural populations (Fig. 6).

Our simulated study demonstrated that our method produced unbiased estimates, yet the accuracy of estimates depends on the data quality (Supplemental Figure S5, S6, S7). Accurate identification of genotypic clusters and transmission sub-clusters has been a historical challenge in TB surveillance. In our study over half (51%) of participants were missing genotypic data, primarily due to culture-negative clinical diagnoses; this proportion is consistent with other studies in the region^46–50^. Since missing genotypic data was not differential by population (48% and 51%, in urban and rural, respectively; Chi-squared p = 0.19), demographic (i.e., 51% for both male and female, Chi-squared p = 0.99 and consistent across age groups, Kruskal–Wallis p = 0.54), or clinical characteristics (i.e., 53% and 51% for HIV-infected and HIV-uninfected, respectively, Chi-squared p value = 0.29), we evaluated the impact of this limitation in our simulation study using a binomial probability. While this revealed that estimates were slightly biased towards homogeneity, this approach assumes genotypic data were missing at random, an assumption that cannot be verified by the empirical data. This assumption would be violated if culture status was differential by genotype. However, to our knowledge, there is no biological rational for coding regions of genes to impact replicative fitness in culture media, and mycobacterial strains from differing lineages share similar growth kinetics.

We also assumed missingness was not differential by population; it is reasonable, for example, that epi-links may be missed among unknown contacts in more densely populated urban area. However, our sensitivity analysis suggests such differences may need to be implausibly large to account for the observed difference in parameter estimates. For instance, despite the situational biases introduced into the model in Fig. 6, upwardly biased estimates in the imperfect rural model (purple) remain sufficiently distinct when assuming perfect surveillance in the urban model (teal).

Our definition of transmission sub-clusters was imperfect. Although we used geocoded addresses to identify primary residences, individuals may be transient or have multiple residences. Our incorporation of epi-link data likely included false transmission events and excluded true transmission events to some unknown degree. Such misclassification will alter the number and size distribution of transmission sub-clusters, but not genotypic clusters. Our approach to evaluate these data limitations was to infer parameters using only genotypic cluster distributions (MIRU-only analysis). MIRU-VNTR is a lower-resolution genotyping method than the more recent whole genome sequencing approach, and generally overestimates cluster distributions^51^. Thus, this assumption makes transmission appear more homogenous (i.e., biases $\hat{k}$ upwards towards homogeneity), and provides a functional upper bound estimate. Analysis of this extreme assumption remained supportive of the high propensity for extensive outbreaks in both populations ($k \ll 1$) yet attenuates the stark differences between the urban and rural population seen when accounting for transmission subclusters (Fig. 2).

All models are a simplified representation of disease transmission and are subject to inherent limitations. Branching process models assume transmission is independent and identically distributed. This assumption would be violated if $\nu$ was correlated among cases within a given transmission chain, which can only be empirically evaluated with knowledge of exact person-to-person transmission events (i.e., transmission chains). Future datasets utilizing higher-resolution molecular techniques such as whole genome sequencing may enable our ability to test this assumption and account for any dependencies between observations. Branching process models also assume the mean susceptibility among individuals remains constant, average susceptibility does not meaningfully decline, and individual infectiousness and susceptibility are uncorrelated. Under this assumption, variation in individual susceptibility, even if unaccounted for, does not influence parameter inference^52^. However, this assumption may be invalid if a substantial proportion of outbreaks occur in clustered pockets of vulnerable sub-populations (i.e., mineworkers^53^) or in scenarios where the depletion of susceptible individuals may meaningfully impact outbreak trajectory (i.e., incarcerated individuals^54^). Recent studies incorporating heterogeneous susceptibility suggest estimates could be both over- and underestimated depending on the network structure and contact distribution patterns^55,56^. Future studies incorporating heterogeneous susceptibility, particularly those in high-burden settings, can extend these findings and deepen our understanding of population-specific transmission dynamics.

Interrupting TB transmission in high-burden settings is fundamental to achieving TB elimination. This analysis developed well-characterized models quantifying TB transmission dynamics in a high-burden setting to estimate the propensity for extensive transmission. The results play a direct role in using surveillance systems to better understand the underlying mechanisms of TB transmission in high-burden populations.

## Supplementary Information

Supplementary Information.

## Data Availability

All code to perform the inference procedure, analysis, and simulations are available at https://github.com/jpsmithuga/UrbanRural_nbbpAnalysis. Data access may be requested by contacting the corresponding author.
