# Supplementary material for: Characterizing tuberculosis transmission dynamics in high-burden urban and rural settings
Source: Sci Rep. 2022 Apr 26;12:6780. doi: 10.1038/s41598-022-10488-2 (PMC9042872; doi:10.1038/s41598-022-10488-2)

Supplemental Materials: Characterizing tuberculosis transmission dynamics in high-burden urban and rural settings

Jonathan P. Smith, PhD, John E. Oeltmann, PhD, Andrew N. Hill, PhD, James L. Tobias, MA, Rosanna Boyd, PhD, Eleanor S. Click, MD, PhD, Alyssa Finlay, MD, Chawangwa Modongo, MD, Nicola M. Zetola, MD, Patrick K. Moonan, DrPH

April 11, 2022

Contents

1 Supplementary Methods **3**
1.1 Modeling Approach 3
1.1.1 Offspring distribution and extracting individual probabilities . 3
1.1.2 Incorporating heterogeneity and the individual reproductive number, ν 4
1.1.3 Inference using cluster size distributions 5
1.1.4 Allowing for sub-clustering 6
1.1.5 Derivation of final probability density function 7
1.1.6 Parameter inference from observed data 8
1.2 SaTScan clustering methods 9
1.3 Burden of secondary transmission 10
1.4 Number of incident cases until first large outbreak 12
1.5 Simulation study and sensitivity analysis 12
1.5.1 Overview 12
1.5.2 Simulating imperfect surveillance 13
1.5.3 Alternative definitions of transmission clusters 14
1.5.4 Alternative models 15

1.6 Additional KOPANYO study details 16
1.6.1 Recruitment details 16
1.6.2 Data sources 16
1.6.3 Sputa collection 17
1.6.4 Genotyping 17
1.7 IPMS data acquisition and hospital linked infection 17

2 Supplemental Figures 20
2.1 Supplementary Figure S1: Common complications arising in TB transmission surveillance. 20
2.2 Supplementary Figure S2: Simulated cluster distributions using three candidate models. 21
2.3 Supplemental Figure S3: Underlying individual heterogeneity in TB transmission, with varying underlying assumptions of ν, by model type. 22
2.4 Supplementary Figure S4: Joint estimates of transmission parameters R and k, by cluster definition (all cases model). 23
2.5 Supplemental Table S5: Comparison of cluster-based and individual-based inference under model parameter assumptions. 24
2.6 Supplemental Table S6: Impact of missing cases on inference of k. . . 25
2.7 Supplemental Figure S7: Assessing the conditional approach to subclustering. 26
2.8 Supplemental Figure S8: Assessing the impact of censorship on parameter inference. 27

1 Supplementary Methods

1.1 Modeling Approach

Branching processes are stochastic, individual-based processes commonly used in epidemiology to model specific chains of transmission. This analysis models transmission using a single-type branching process, also known as a discrete time Galton-Watson process. This method is the most well-studied and validated approach to branching processes. Each infected individual is associated with a fixed length time interval known as a generation; at the end of each generation, each individual in the generation will have produced a random number of secondary infections (or "offspring"), denoted Z, drawn from some probability distribution.

1.1.1 Offspring distribution and extracting individual probabilities

The offspring distribution is the probability distribution for the observed number of secondary cases caused by each individual infectious case, Z (i.e. $p_z = P(Z = z)$ for $z = 0, 1, 2, 3, \ldots$). A fundamental tool in the analysis of any branching process model is the probability generating function (pgf) of the offspring distribution. In a branching process framework, the pgf is a mathematical tool to study the sequence of probabilities and contains all the information needed to recover the probabilities associated with each Z value. The pgf in branching processes can generally be expressed as:

$$G_Z(s) = p_0 + p_1 s + p_2 s^2 + p_3 s^3 + \cdots + p_z s^z + \cdots = \sum_{z=0}^{\infty} p_z s^z$$

Where s is a dummy variable whose powers serve as a placeholder to recover the probabilities associated with Z and facilitate the use of high-order derivatives in their recovery, such that:

$$P(Z = z) = p_z = \frac{1}{z!} \frac{d^z G_Z(s)}{ds^z}\bigg|_{s=0}$$

Where $z = 0, 1, 2, 3, ...$ Here, $\frac{d^z G_Z(s)}{ds^z}$ is the zth derivative of $G_z(s)$ evaluated at $s = 0$ and $1/z!$ is a normalizing constant. This affords practical extraction of any probability of z. For instance, consider the simple example of extracting the probability that $Z = 3$, we simply we take the third derivative, evaluate at $s = 0$, and normalize by $1/3!$:

$$G'''_Z(s) = (3 \times 2 \times 1)p_3 + (4 \times 3 \times 2)p_4 s + \cdots + z(z-1)(z-2)p_z s^{z-3} + \cdots$$
$$G'''_Z(0) = (3!)p_3$$
$$\frac{1}{3!}G'''_Z(0) = p_3$$

1.1.2 Incorporating heterogeneity and the individual reproductive number, ν

Traditional compartmental differential-equation ("SIR") models incorporate heterogeneity by assigning a vector of known covariates to each individual (i.e., gender, HIV status, etc.) and categorizing them into groups; homogeneous mixing and constant infectious periods are assumed for individuals with the same group. Our approach contrasts in that it incorporates unknown and unidentifiable factors that may attribute to heterogeneity by assuming the number of secondary cases resulting from each person, ν, is drawn from a continuous probability distribution with mean R.[3] ν encodes the entire infectious history of each individual and intrinsically accounts for all known and unknown individual characteristics that might modulate the number of secondary cases for an individual.[3] We can account for different degrees of heterogeneity by specifying a distribution of ν; for instance, keeping R constant at 0.90, the probability density of ν is markedly different assuming a geometric distribution vs gamma distribution (with dispersion $k = 0.75$) is shown in Figure M1.

Figure M1: Influence of distributional assumption on ν

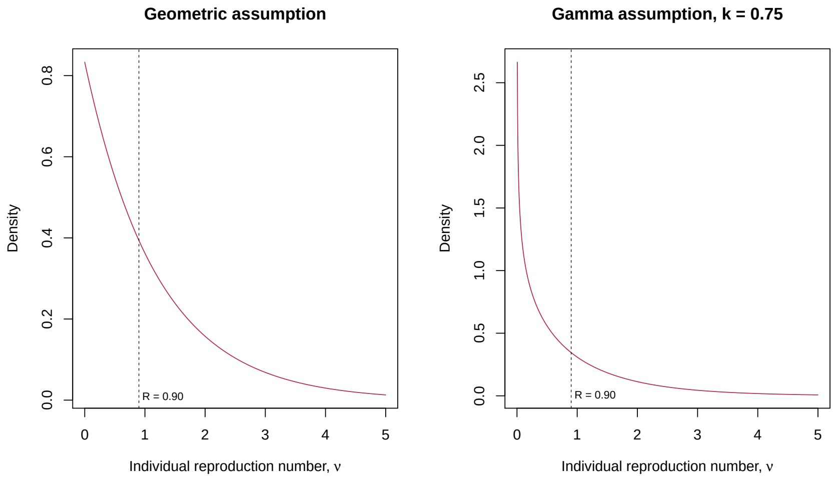

ν is a theoretical expectation drawn from a continuous distribution that can take on any non-integer real value, however the observed number of secondary cases must be an integer, and is a result of ν and demographic stochasticity. By modeling the distribution of Z conditional on an observed value of ν as a Poisson process, we have $Z \sim Poi(\nu)$. In this analysis, we assume the number of cases for each individual, ν, is drawn from an underlying gamma distribution with mean R and dispersion parameter k. The observed Z values are a poisson-mixed gamma process resulting in a negative binomial distribution of Z. This mixture is shown as:

$$\begin{aligned}P(Z=z) =& \frac{k^k}{\Gamma(k)R^k}\int_0^\infty \frac{e^{-\nu}\nu^z}{z!}\nu^{k-1}e^{-k\nu/R}d\nu \\ =& \frac{k^k}{\Gamma(z+1)\Gamma(k)R^k}\int_0^\infty \nu^{k+z-1}e^{-\nu-\nu/R}d\nu \\ =& \frac{\Gamma(z+k)}{\Gamma(z+1)\Gamma(k)}\left(\frac{k}{R+k}\right)^k\left(\frac{R}{R+k}\right)^z\end{aligned}$$

Which is a negative binomial distributed offspring distribution. The variance of the negative binomial distribution is $R(1+R/k)$, thus by its position in the denominator of the variance k quantifies the degree of overdispersion in the distribution (i.e., more individual heterogeneity in secondary cases). When $k=1$, the variance is $R(1+R)$ and the negative binomial distribution reduces to the geometric distribution. As $k \to \infty$, the variance reduces to R and the negative binomial converges to the Poisson distribution. The negative binomial distribution has the following probability generating function (pgf):

$$G_Z^{NB}(s) = \sum_{z=0}^{\infty}\frac{\Gamma(z+k)}{\Gamma(z+1)\Gamma(k)}\left(\frac{k}{R+k}\right)^k\left(\frac{R}{R+k}\right)^z s^z = \left(1+\frac{R(1-s)}{k}\right)^{-k} \quad (1)$$

1.1.3 Inference using cluster size distributions

Transmission chains give rise to transmission clusters, thus there is a mathematical relationship between the generating function of Z and the generating function of total cluster sizes, Y, defined as $G_Y(s)$. The recursive relationship that defines the total number of secondary infections in a single transmission chain, including the index case, can be defined as:[4]

$$G_Y(s) = sG_Z((G_Y(s))$$

This relationship is intuitive with small clusters. The probability that a single individual case transmits no secondary infections ($Z = 0$) is equal a cluster reaching a final of size one, i.e., $P(Z = 0) = P(Y = 1)$. If an infectious case results in only one secondary case, there is only one possible chain of transmission: the index case results in one secondary case, which results in zero secondary cases. Thus, $P(Y = 2) = P(Z = 1)P(Z = 0)$. When a cluster of size three arises, there are two possible chains of transmission: (1)the index case transmits to two people who both transmit to zero people, or (2) the index case transmits to one person, who transmits to another, who does not transmit). Thus, $P(Y = 3) = P(Z = 2)P(Z = 0)^2 + P(Z = 1)^2P(Z = 0)$. We can extend this relationship to any cluster of size Y using the same calculus described in section 1.1.1 [6]:

$$P(Y = y) = \frac{1}{y!}\frac{d^y G_Y(s)}{ds^y}\bigg|_{s=0}$$

1.1.4 Allowing for sub-clustering

A common manipulation of branching processes theory is the multiplication of generating functions, i.e., $G_z(s)^i$ represents all the possible ways i cases can result in z secondary cases. With this in mind, we can adapt branching processes to cluster size distributions under certain constraints. In our case, for a given MIRU-VNTR cluster of size Y containing n transmission sub-clusters, there are exactly $Y - n$ transmission events, regardless of the sequence of transmission.[1] For instance, Figure M2 shows a genotypic cluster of size nine with two transmission sub-clusters will always have seven transmission events ($Y - n = 9 - 2 = 7$).

Figure M2: Visualization of sub-clustering

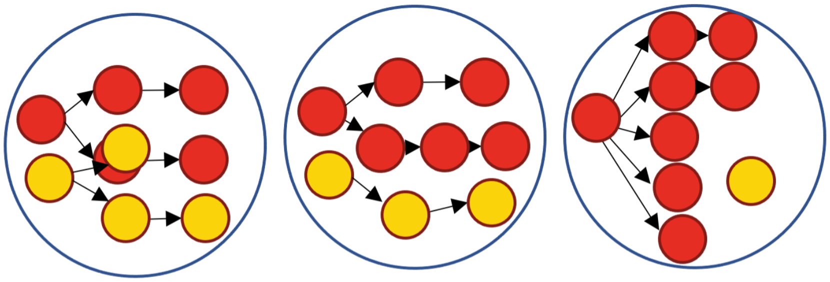

Where blue outlines represent a genotypic cluster and red and yellow circles represent distinct transmission sub-clusters; black arrows represent resulting secondary cases

from transmission. Following the convention above, the probability that n subclusters result in $y-n$ secondary cases can be represented as:

$$\frac{1}{(y-n)!}\frac{d^{y-n}G_Z(s)^y}{ds^{y-n}}\bigg|_{s=0}$$

However, this is a purely mathematical consequence that is akin to the probability of randomly drawing n integers from an offspring distribution and having them sum to $y-n$. In infectious disease transmission, the sequence of events must be biologically relevant and only a subset of permutations, equal to n/y [1], are biologically plausible transmission chains. A simplified example of this concept is depicted in Figure M3 for a transmission cluster of size three with one index case (i.e. $y=3$ and $n=1$):

Figure M3: Visualization of cyclic permutations

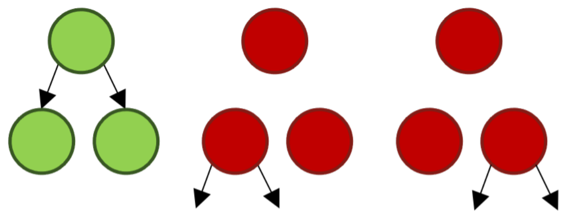

While all three cyclic permutations satisfy the $y-n$ constraint, only one of these cyclic permutations is a biologically plausible transmission chain (green); assuming they are equally likely to be observed, $n/y = 1/3$. To account for this, we normalize the generalized probability by (n/y):

$$P(Y=y|n) = \left(\frac{n}{y}\right)\frac{1}{(y-n)!}\frac{d^{y-n}G_Z(s)^y}{ds^{y-n}}\bigg|_{s=0}$$

1.1.5 Derivation of final probability density function

Our primary assumption is the ν is gamma distributed with mean R and dispersion parameter k. As shown in Equation 1 (section 1.1.2), this results in a negative binomial offspring distribution with identical mean R and dispersion k. For clarity, we can let $p = R/(R+k)$ and rewrite the negative binomial pgf described as:

$$G_Z^{NB}(s) = \left(\frac{1-p}{1-ps}\right)^k = (1-p)^k(1-ps)^{-k}$$

Recalling the property of multiplying generating functions, we can write $G_Z(s)^y = (1-p)^{ky}(1-ps)^{-ky}$. We can then use Taylor expansion (generally noted as $f(x) = (1+x)^a$) of $G_Z(s)^y$ to facilitate the derivation of the final cluster size:

$$\begin{aligned} G_Z^{NB}(s)^y =&(1-p)^{ky}(1-ps)^{-ky} \\ =&(1-p)^{ky}\Bigg(1 + kyps + \frac{ky(ky+1)}{2!}p^2s^2 + \cdots \\ &+ \frac{ky(ky+1)\cdots(ky+(y-n)-1)}{(y-n)!}p^{y-n}s^{y-n} + \cdots\Bigg) \end{aligned}$$

Following notation in previous sections, the coefficient of s^{y-n} can also be found by taking the $(y-n)^{th}$ derivative evaluated at zero:

$$\begin{aligned} \frac{1}{(y-n)!}\frac{d^{y-n}G_Z^{NB}(s)^y}{ds^{y-n}}\Bigg|_{s=0} =&\frac{(ky+y-n-1)!}{(ky-1)!(y-n)!}(1-p)^{ky}p^{y-n} \\ =&\binom{ky+y-n-1}{y-n}(1-p)^{ky}p^{y-n} \end{aligned}$$

This only holds when k is an integer, however we can extend this to any real number k by noting that:

$$\frac{ky(ky+1)+\cdots+(ky+y-n-1)}{(y-n)!} = \frac{\Gamma(ky+y-n)}{\Gamma(ky)(y-n)!}$$

Substituting $p = R/(R+k)$ with algebraic manipulation and recalling that only an n/y proportion of the coefficient of s^{y-n} result in valid transmission sequences, we can express the final probability of a cluster initiating with n index cases and going extinct at exactly y cases as:

$$P(Y = y|n) = \frac{n}{y} \times \frac{\Gamma(ky+y-n)}{\Gamma(ky)(y-n)!}\frac{(\frac{R}{k})^{y-n}}{(1+\frac{R}{k})^{ky+y-n}} \tag{2}$$

Where $y = 1, 2, 3\ldots$ and $y \geq n$.

1.1.6 Parameter inference from observed data

To infer parameters R and k from the observed data we used maximum likelihood estimation (MLE). We considered clusters as wither wholly observed or censored.

As defined in the manuscript, clusters of at least size five with an incident case arising within two years were considered censored. We included censored clusters in the likelihood by calculating the probability that a cluster is at least size Y.[2] The likelihood of parameters R and k when a fully observed genetic clusters containing n transmission sub-clusters and b censored genetic clusters containing n transmission sub-clusters can we expressed as:

$$L(R, k|a, b) = \prod_{y=1}^{\infty} \prod_{n=1}^{y} P(Y = y|n)^{a_{y,n}} \prod_{y=1}^{\infty} \prod_{n=1}^{y} P(Y \geq y|n)^{b_{y,n}}$$

Where $P(Y = y|n)$ is the negative binomial probability defined in Equation (2) and $P(Y \geq y|n) = 1 - \sum_{i=1}^{y-1} P(Y = y|n)$

1.2 SaTScan clustering methods

Primary residential address, work place address at diagnosis, and address of social gathering venues of patients were obtained through patient interview. All addresses were verified by site visit geotagging, or through a reference layer created by manually relocating addresses in satellite imagery by using OpenStreetMap (`http://www.openstreetmap.org`), Google Maps, and ArcGIS (Environmental System Research Institute, `https://www.esri.com`) online geocoding services. WGS 84 projection system latitude and longitude coordinates (with 1.1-meter precision) were exported for each address.

We used SaTScan (`https://www.satscan.org`) to identify geographic areas with a larger-than-expected rate of unique genotype clusters. We also used data for all other culture-positive TB patients reported during the study as the background rate. All individual MIRU-VNTR results were assigned to the corresponding geocoordinates of the patient's residence. Each unique MIRU-VNTR result was then scanned separately, applying a purely spatial analysis, in which the number of events in an area was assumed to be Poisson distributed to generate circular zones of various sizes up to a maximum radius maximum cluster size up to the entirety of Gaborone and Ghanzi. A log-likelihood ratio was calculated for each zone in comparison with all possible zones, with the maximum likelihood ratio representing the zone most likely to identify statistically significant spatial concentrations for each MIRU-VNTR result. Thus, by definition, localized transmission was characterized by genotypic and spatial clustering. A Monte Carlo simulation with 9,999 repetitions was used to determine the distribution of the scan statistic under the null hypothesis of spatial

randomness; significant spatial clusters were chosen by using an α of $p < 0.05$. No duplicative case counting occurred. The purpose of the spatial scan was to characterize each patient (based on residence) for a dichotomous outcome: member of a localized transmission event or not.

1.3 Burden of secondary transmission

To estimate the proportion of transmission attributable to a set proportion of infectious cases (Figure 5 in the main text), we follow Lloyd-Smith et al. [3] who propose a distribution for describing transmission based from the distribution of the individual reproduction number ν:

$$F_{\text{trans}}(x) = \frac{1}{R} \int_0^x u\, f_\nu(u)\, du$$

This gives the cumulative distribution function (CDF) in terms of the individual reproduction number density f_ν, as the proportion of all transmission due to infectious individuals with reproduction number $\nu < x$. In the specific case when ν is gamma distributed with shape parameter $k > 0$ and rate parameter k/R_0, where R_0 is the mean (expected) individual reproduction number, we have:

$$f_\nu(u) = \frac{k^k}{R^k\, \Gamma(k)} u^{k-1} e^{-ku/R}$$

It follows that

$$\begin{aligned} f_{\text{trans}}(x) &= F'_{\text{trans}}(x) \\ &= \frac{1}{R} x f_\nu(x) \\ &= \frac{k^k}{R^{k+1}\, \Gamma(k)} x^k e^{-kx/R} \\ &= \frac{k^{k+1}}{R^{k+1}\, \Gamma(k+1)} x^k e^{-kx/R} \\ &\sim \text{Gamma}(k+1, k/R) \end{aligned}$$

The rate parameter remains the same and the shape parameter increases by 1.
To calculate t_p, the expected proportion of transmission due to the most infectious $100p\%$ of cases, we first find the $(1-p)$th centile of the individual reproduction number, $x_p = F_\nu^{-1}(1-p)$, then calculate $t_p = 1 - F_{\text{trans}}(x_p)$. As both random variables are

gamma distributed, this is implemented in R via the qgamma (F^{-1}) and pgamma (F) functions. We apply this to the Kopanyo urban and rural estimates for R and dispersion (scale) parameter k.

The relationship between p and t_p
Parameterizing by x_p, and noting that $p = 1 - F_\nu(x_p)$, the first derivative is

$$\frac{dt_p}{dp} = \frac{dt_p/dx_p}{dp/dx_p} = \frac{-F'_{\text{trans}}(x_p)}{-F'_\nu(x_p)} = \frac{R^{-1}x_p f_\nu(x_p)}{f_\nu(x_p)} = \frac{x_p}{R} > 0$$

which confirms that t_p is an increasing function of p. When $x_p = 0$, $(p, t_p) = (1, 1)$ at which the point the derivative is zero (horizontal tangent). Similarly, when $x_p = +\infty$, $(p, t_p) = (0, 0)$ at which the point the derivative is infinite (vertical tangent).
The second derivative is negative implying concavity of the graph of t_p as a function of p.

$$\frac{d^2t_p}{dp^2} = \frac{d}{dx_p}\left(\frac{dt_p}{dp}\right) \times \frac{dx_p}{dp} = \frac{1}{R_0} \times \frac{1}{dp/dx_p} = -\frac{1}{Rf_\nu(x_p)} < 0$$

1.4 Number of incident cases until first large outbreak

To estimate the number of incident cases until the first outbreak of size Y (Figure 4C in the main text), we simulated 500 surveillance systems, each with 2000 transmission chains beginning with a single introduction (assumed to be reactivation of latent TB infection or importation (i.e., migration)). For each infectious individual, ν was drawn from a gamma distribution with mean R and k specified by the inference procedure of the respective urban and rural models. Each individual was infectious for only one generation and the chain continued until extinction. The total transmission cluster size was the sum of all generations in the transmission chain. The first incident case to reach the specified Y value was recorded. Y values were arbitrarily chose to reasonably represent epidemiologically relevant and realistically large outbreaks.

1.5 Simulation study and sensitivity analysis

1.5.1 Overview

Using our branching process framework, we simulated data to model underlying TB transmission in high-burden surveillance systems under values of R and k inferred from each model. For these purposes, individual-level transmission "chains" are defined as the exact sequence of underlying transmission events (i.e., transmission trees) originating from a single index case. Transmission chains are considered to originate by the sporadic activation of latent TB or by the introduction of an infectious individual into the population (i.e., migration). A transmission "cluster" is defined as the final chain size, including the index case and all cases from all subsequent generations (i.e., secondary, tertiary, etc.) in the chain. For the purposes of this analysis, an index case with no secondary transmission is considered a "cluster" of size one1. Each individual branching process originated with a single index case and continued until extinction. A simulated surveillance system consisted of N individual underlying true transmission chains. Unless otherwise stated, we simulated 500 surveillance systems, each containing 2000 transmission chains. Final transmission cluster sizes (Y values) were the sum of each transmission chain, including the index case. Thus, under perfect surveillance, simulated cluster data were a simple vector of transmission cluster sizes and obscured all information on individual transmission events.

1.5.2 Simulating imperfect surveillance

We modeled several common real-world limitations affecting cluster size data in TB surveillance (Supplementary Figure S1). Incomplete case ascertainment was simulated in a two-step process to emulate TB surveillance practices closely as possible. First, each case within the chain was independently observed with binomial probability p_1, representing the ability of the surveillance system to ascertain cases passively (i.e., $p_1 = 1$ indicates perfect observation). While this approach simulates missing cases by passive surveillance, many public health systems attempt to identify other and otherwise undiagnosed cases in the chain of transmission to some degree, either by contact tracing, patient information, or other means. This may bias the distribution of cluster sizes by shifting the distribution to the right; small clusters or individual cases are more likely to be wholly unobserved than large clusters. To simulate active case finding, all missing cases in chains with at least one case identified through passive surveillance were re-evaluated with probability p_2. After evaluation of p_1 and p_2, chains may be "broken" into two or more observed pseudo-clusters depending on the position of missing cases (Supplemental Figure S1C). Censored chains were incomplete chains due to the sampling time frame and represent ongoing transmission clusters at the time of data collection (Figure 1D). Each chain could be censored with binomial probability p_{cens}, set from the empirical data (12 percent and 6 percent in the urban and rural models, respectively). The generation of spread in the individual branching process where censoring began was randomly selected from all the generations in the chain using a uniform distribution. The generation selected for censoring and all subsequent generations were not observed regardless of p_1 or p_2. Data from clusters after the censoring process were a matrix of two columns, the cluster size and a dichotomous variable indicating censored status (censored or not censored).

MIRU-VNTR clusters containing multiple transmission sub-clusters result in a combined single cluster of size y with n transmission sub-clusters. Sub-clustering was simulated by first determining the proportion of clusters in a surveillance system that overlap, p_over, derived from the empirical models (12 percent and 19 percent in the urban and rural models, respectively). Simulating overlap was iterative; in each iteration the process randomly drew and merged n clusters from the surveillance system, resulting in a final cluster size of $Y = \sum_n y_n$ with n sub-clusters. The number of sub-clusters merged in each iteration (n) was drawn from a Poisson distribution with λ set to the mean number of transmission sub-clusters in the observed data, allowing for simulations to more accurately follow empirical estimates of the number of index cases identified from overlapping cluster. The iterative process repeated until the

proportion of chains in the surveillance system designated by p_{over} was satisfied. Final simulated transmission chains were subject to any combination of these scenarios. Final imperfect simulated data was a matrix containing the imperfect cluster size, the censorship status of each cluster, and the number of sub-clusters in each cluster. "Perfect observation" was an ideal scenario where all cases in the transmission chain were perfectly observed and is the reference for the inference procedure. All simulations and calculations were completed using R statistical programming.

1.5.3 Alternative definitions of transmission clusters

We considered three alternatives transmission cluster definitions to the SaTScan approach (Figure M4); (1) a next-nearest neighbor approach using residence plots (parcels); (2) a one-kilometer approach solely using Euclidean distance; and (3) a MIRU-only definition. The next nearest neighbors method has been shown to reasonably approximate recent transmission. [5] This approach considers an index case as the first culture-positive pulmonary TB case in a household; the index case's geocoded and validated primary residence was used to identify immediate neighbors and next-nearest neighbors (two parcels away). Participants with the same genotype within the same two-parcel space were considered a transmission cluster. In addition, cases with the same genotype but part of a separate two-parcel contact are also considered in the same transmission cluster. This assumes there is an unknown epidemiologic link between households. The one-kilometer approach simply considers all cases with the same genotype residing within one kilometer as part of a transmission cluster. These two definitions are more geographically restrictive than SaTScan-based clustering and likely underestimate the true size of underlying transmission clusters. Lastly, we assumed MIRU-VNTR clusters were themselves wholly observed transmission clusters, which is known to overestimate transmission. By including these intentionally conservative and liberal secondary definitions we assess a range of plausible bounds for parameter inference.

Figure M4: Alternative sub-cluster definitions

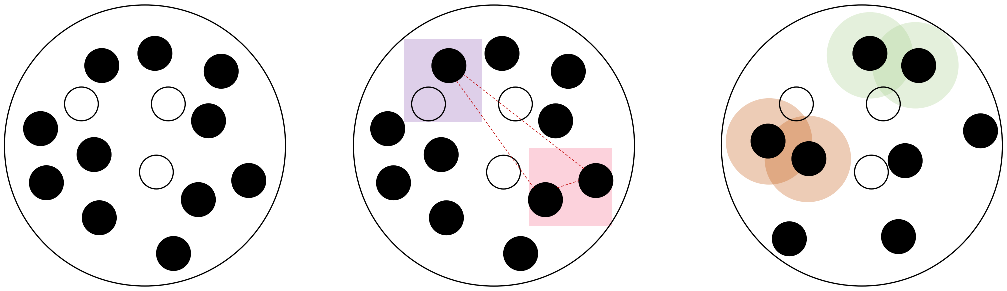

MIRU Only

Only uses MIRU (black circles) to define transmission clusters. Here, a cluster of size of y=11.

MIRU + Next Nearest Neighbor

Cases with the same MIRU and within the same 2-neighbor space (red shade) are still considered a sub-cluster. In addition, cases with the same MIRU but part of another 2-household contact (purple shade) are also considered part of the same sub-cluster. This assumes there is an unknown link between households (red dotted line). Here, this results in a one sub-cluster of size y=3 and eight subclusters of size y=1

MIRU + One Kilometer

Cases with the same MIRU and one kilometer of each other were considered a transmission cluster. This figure represents a genotypic cluster of size 8 with two transmission sub-clusters of size $y = 2$ and four isolated cases.

1.5.4 Alternative models

Homogenous model: We varied the distribution of ν to assess the appropriateness of the gamma-distribution assumption. If we assume there is no underlying mechanism of individual heterogeneity, then differences in the number of secondary cases between individuals is solely attributed to stochasticity and $\nu = R$, thus $Z \sim POI(R)$. Under the assumption of homogeneous transmission, Z is Poisson distributed and the distribution of final cluster sizes, Y, containing n sub-clusters follows a Borel-Tanner distribution:[2]

$$P(Y = y|n) = \frac{ny^{y-n-1}R^{y-n}e^{-yR}}{(y-n)!}$$

Where $n = 1, 2, 3 \ldots$ and $y \geq n$.

SIR-Type model: As a more realistic alternative, we approached the assumptions made by traditional SIR models. If we assume homogeneous mixing with a constant infectious period, ν is exponentially distributed. Under this "SIR-type" assumption,

Z is geometrically distributed and the distribution of final cluster sizes, Y, containing n sub-clusters follows a Lagrangian generalized negative binomial distribution:[2]

$$P(Y = y|n) = \frac{n}{2y - n}\binom{2y-n}{y-n}\frac{R^{y-n}}{(1+R)^{2y-n}}$$

Where $n = 1, 2, 3\ldots$ and $y \geq n$.

1.6 Additional KOPANYO study details

The below sections provide brief details on patient recruitment, data acquisition, and other pertinent factors related to the Kopanyo study. A full protocol detailing the exact procedures can be found here [7]: `https://bmjopen.bmj.com/content/6/5/e010046`

1.6.1 Recruitment details

Participants were enrolled from August 2012 to March 2016 by study staff. Patients were recruited into the study at the time of enrollment; all patients given a diagnosis of TB were eligible. Participants were recruited from TB clinics and directly observed treatment centers in greater Gaborone and Ghanzi District. Only patients receiving TB treatment for more than 14 days before study screening, incarcerated persons, or those who did not consent were excluded from the study.

HIV status was determined for all enrolled participants. We offered HIV testing to all participants who did not have documented HIV test results or had negative test results from greater than 12 months before enrollment.

1.6.2 Data sources

Study staff obtained behavioral, clinical, and demographic information from both abstracting medical records and from conducting standardized interview at enrollment. Location data, including residential and work addresses, and locations of social gathering venues, were initially obtained through patient interview then verified by site visit geotagging. For any venue unable to be verified by site visit, coordinates were obtained manually via relocating addresses in satellite imagery. WGS 84 projection system latitude and longitude coordinates (with 1.1-m precision) were exported for each location.

1.6.3 Sputa collection

At least one expectorated sputum sample was obtained from each enrolled patient. If patients were unable to produce enough sputum or unable to produce high-quality sputum, we performed inhaled nebulized hypertonic saline solution induction. Sputa were decontaminated by using the N-acetyl-L-cysteine and NaOH method with a final concentration of 1% NaOH, and then inoculated into 1 Mycobacterial Growth Indicator Tube (MGIT; Becton Dickinson, `https://www.bd.com`). MGIT cultures were incubated at 35°C – 37°C in the MGIT960 instrument (Becton Dickinson) for < 6 weeks. MGIT cultures scored as positive were examined by microscopy and Ziehl-Neelsen staining to identify acidfast bacilli. SD. The Bioline TB Ag MPT64 Rapid Test (Abbott, `https://www.globalpointofcare.abbott/en/product-details/sd-bioline-tb-ag-mpt64-rapid.html`) was used to identify the M. tuberculosis complex. Cultures positive for acid-fast bacilli but with negative TB Ag MPT64 results were classified as nontuberculous mycobacteria. Cultures with evidence of both Mycobacterium species and other potential contaminating species were redecontaminated by using the standard method described above. Drug susceptibility testing (DST) for first-line anti-TB drugs was performed by using MGIT DST. Susceptibility was set at 0.1 $\mu g/mL$ for isoniazid and 1.0 $\mu g/mL$ for rifampin. We used DST with Lowenstein-Jensen medium in instances for which MGIT DST results were not available.

1.6.4 Genotyping

Following standard procedures, the first culture isolate per patient was genotyped using 24-locus mycobacterial interspersed repetitive units – variable number of tandem repeats (MIRU-VNTR) and standardized methods (Genoscreen, `https://www.genoscreen.fr`). MIRU-VNTR results with >1 copy number at >1 loci (i.e., double alleles), or with missing or indeterminate copy number at any locus, were considered noninterpretable for cluster assignment and were excluded from analysis. Two or more patient isolates that had valid, complete, and matching MIRU results were classified as a genotype cluster.

1.7 IPMS data acquisition and hospital linked infection

For all participants, we abstracted all discrete clinical encounters occurring between March 1, 2004 and December 31, 2018 from Botswana's Ministry of Health's Integrated Patient Management System (IPMS), a universal, centralized electronic medical record (EMR) system. IPMS contains detailed information for all inpatient

and outpatient visits to any public or private healthcare facility in the country (hospital, clinic, health post, etc.) and can be accessed from any health facility using the system. IPMS entries document the specific location, type (i.e., inpatient or outpatient), specific hospital ward (if inpatient), date of visit, and health coding for each discrete clinical encounter, including dental care, diagnostics, laboratory, pharmacy, radiography, and hospitalization. IPMS data were linked to the Kopanyo dataset by OMANG number. For participants with missing or incorrect OMANG numbers, name, gender, and date of birth were used to identify the participants' IPMS data.

We overlaid IPMS data with clinical TB data (date of diagnosis, treatment initiation, etc) to identify potential transmission at healthcare facilities. We first defined any participants with the same genotype that were present at the same healthcare facility at the same time as an overlapping event. We then considered clinical TB information to identify potential transmission. We considered possible transmission two separate ways. First, consistent with many contact tracing efforts, we defined a patient's infectious period as 3 months (93 days) prior to diagnosis and 14 days after the initiation of treatment. A patient was considered infectious during any inpatient or outpatient visit during this time. If an inpatient visit spanned the 93-day threshold (i.e., a patient was admitted 95 days prior to diagnosis and discharged 85 days prior to diagnosis), the infectious period was extended to incorporate the entire duration of the inpatient stay. Any patient with the same genotype overlapping with another patient at a healthcare facility during their infectious period was considered an epidemiological link ("epi-link") and included in a transmission subcluster per the methods described in the main text regardless of time between the overlap and progression to clinical disease. Second, if two or more patients with the same genotype overlapped at a healthcare facility before diagnosis, and were all later diagnosed within two years, we considered this an epi-link as it represents possible infection from an unknown source.

Given the natural history of TB, participants that were concomitantly diagnosed during an overlapping event were assumed to have acquired infection in the community and not considered an epi-link. In addition, overlapping events where all participants (or all but one participants) were diagnosed before the event occurred were excluded as transmission had already occurred prior to the visit.

References

[1] Niels Becker. On parametric estimation for mortal branching processes. *Biometrika*, 61(3):393–399, 1974.

[2] C. P. Farrington, M. N. Kanaan, and N. J. Gay. Branching process models for surveillance of infectious diseases controlled by mass vaccination. *Biostatistics*, 4(2):279–295, 04 2003.

[3] J. O. Lloyd-Smith, S. J. Schreiber, P. E. Kopp, and W. M. Getz. Superspreading and the effect of individual variation on disease emergence. *Nature*, 438(7066):355–359, November 2005.

[4] Charles J Mode and Candace K Sleeman. *Stochastic Processes in Epidemiology.* World Scientific, 2000.

[5] Patrick Moonan, Nicola Zetola, James Tobias, and Joyce Basotli. A neighbor-based approach to identify tuberculosis exposure, the kopanyo study. *Emerging Infectious Diseases*, 26, 2020.

[6] Ping Yan. *Distribution Theory, Stochastic Processes and Infectious Disease Modelling*, pages 229–293. Springer Berlin Heidelberg, Berlin, Heidelberg, 2008.

[7] N M Zetola, C Modongo, P K Moonan, E Click, J E Oeltmann, J Shepherd, and A Finlay. Protocol for a population-based molecular epidemiology study of tuberculosis transmission in a high hiv-burden setting: the botswana kopanyo study. *BMJ Open*, 6(5), 2016.

2 Supplemental Figures

2.1 Supplementary Figure S1: Common complications arising in TB transmission surveillance.

Filled circles represent observed individuals and grey filled circles represent unobserved; arrows represent true underlying transmission events. Dotted circles represent final cluster sizes. A) Perfect surveillance, all cases originating from a single index case are completely observed; B) Incomplete ascertainment with i missing cases results in a cluster size of $Y - i$; C) "Broken chains" occurs when the position of the missing case in the chain results in j pseudo-clusters, which themselves may be subject to incomplete ascertainment; D) Censored chains are ongoing chains at the time of data collection; E) Sub-clustering results when n chains are unable to be disentangled, resulting in a single cluster of size y with n sub-clusters.

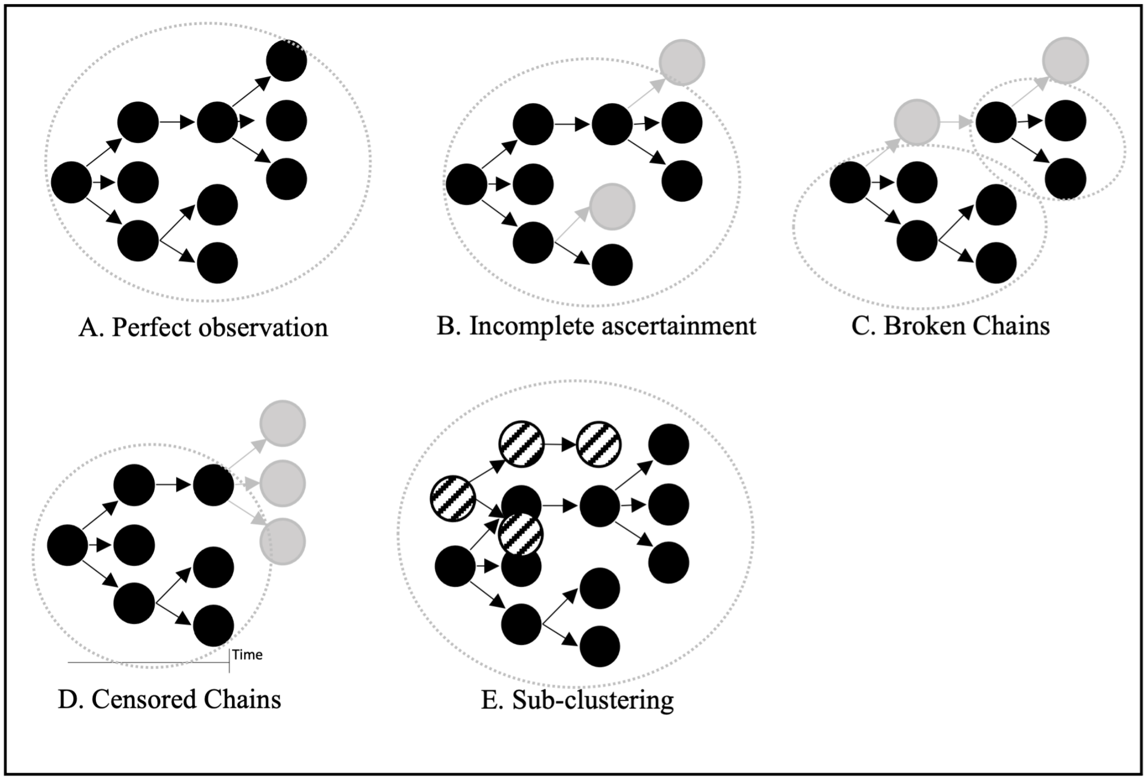

2.2 Supplementary Figure S2: Simulated cluster distributions using three candidate models.

We simulated TB transmission in each population (rows) using the three candidate models (columns). Results from 500 simulated surveillance systems each with 2000 transmission chains.

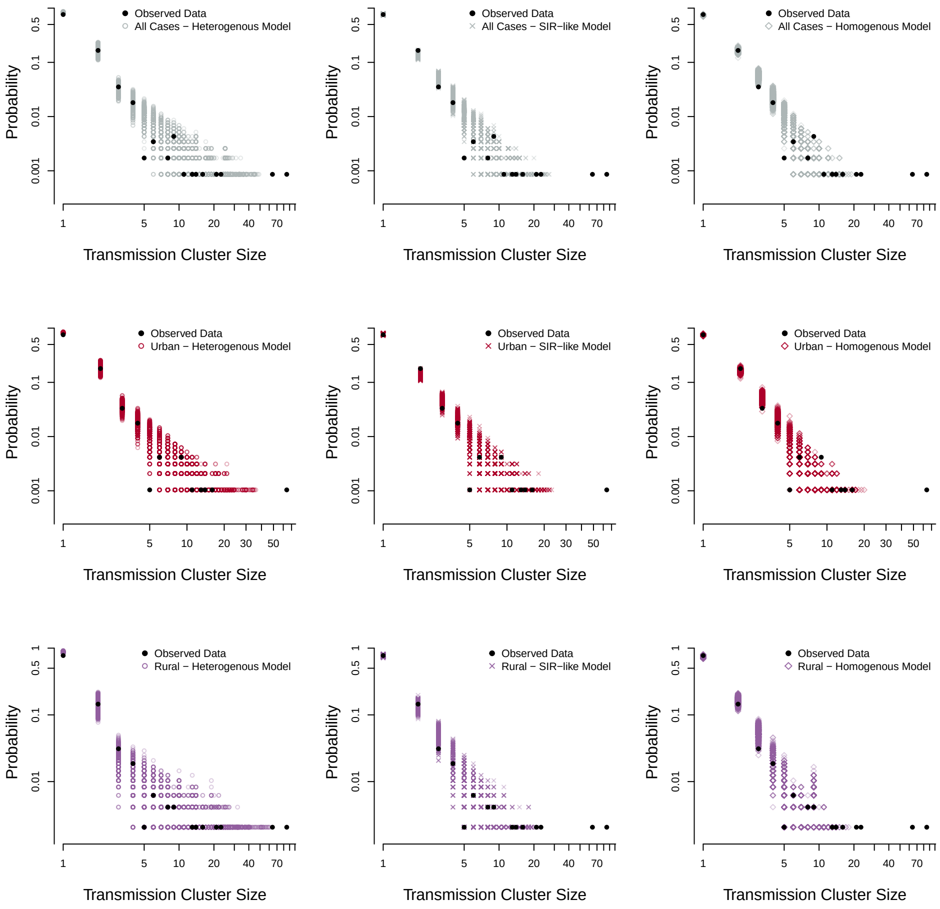

2.3 Supplemental Figure S3: Underlying individual heterogeneity in TB transmission, with varying underlying assumptions of ν, by model type.

Homogeneous model: $\nu = R$. The SIR-type model: ν was exponentially distributed with mean R. The heterogeneous model allowed k to be a free parameter and accounted for an unknown degree of heterogeneity; ν is gamma distributed with mean R and dispersion k. (A) and (B) Probability of observing a large outbreak by model type; (C) and (D) Probability density of expected number of secondary cases for each individual (i.e., underlying individual reproductive number, ν).

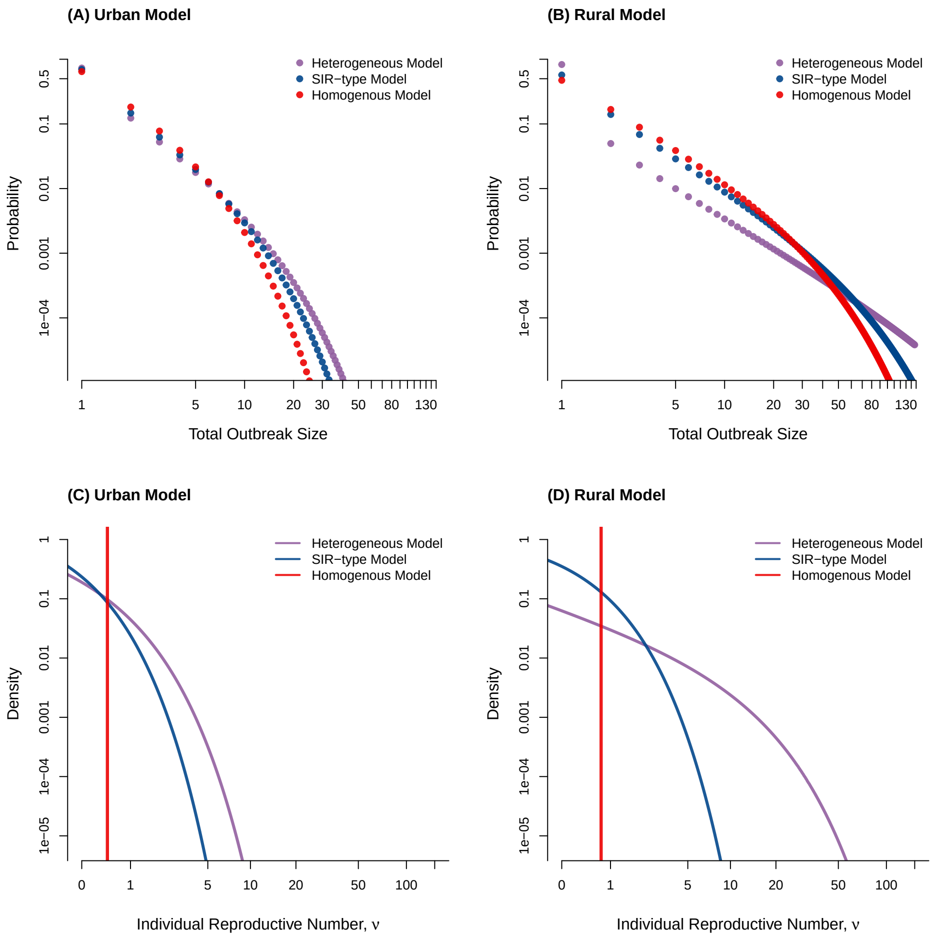

2.4 Supplementary Figure S4: Joint estimates of transmission parameters R and k, by cluster definition (all cases model).

We jointly estimated R and k in the total population using four transmission cluster definitions. Our primary cluster definition used a spatial scan statistic to determine statistically significant transmission clusters. Two alternative definitions ("Next Nearest Neighbor" and "One Kilometer") underestimated the size of transmission, and MIRU-only clusters overestimated the size of transmission clusters.

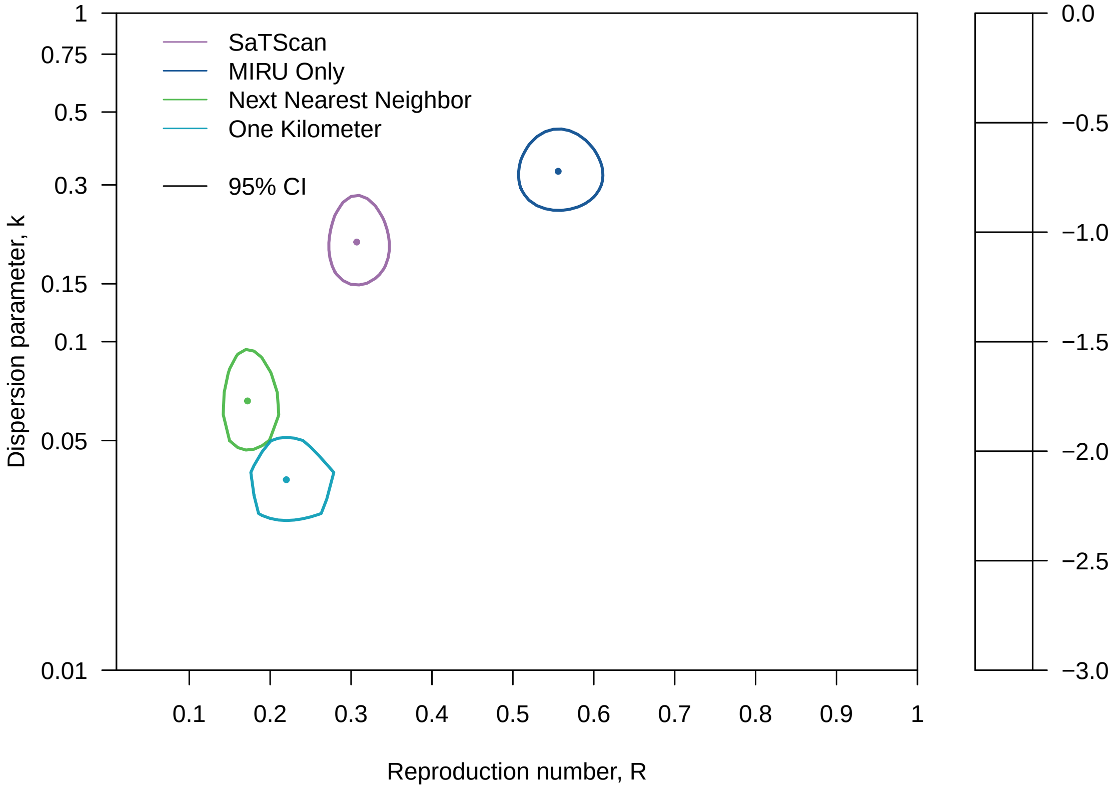

2.5 Supplemental Table S5: Comparison of cluster-based and individual-based inference under model parameter assumptions.

We simulated 500 surveillance systems, each containing 2000 individual transmission chains assuming perfect surveillance for each model. We then used traditional maximum likelihood (MLE) methods and the cluster based MLE methods to evaluate performance. Simulated true underlying parameters were $R = 0.44$ and $k = 0.48$ in the urban model and $R = 0.75$ and $k = 0.08$ in the rural model.

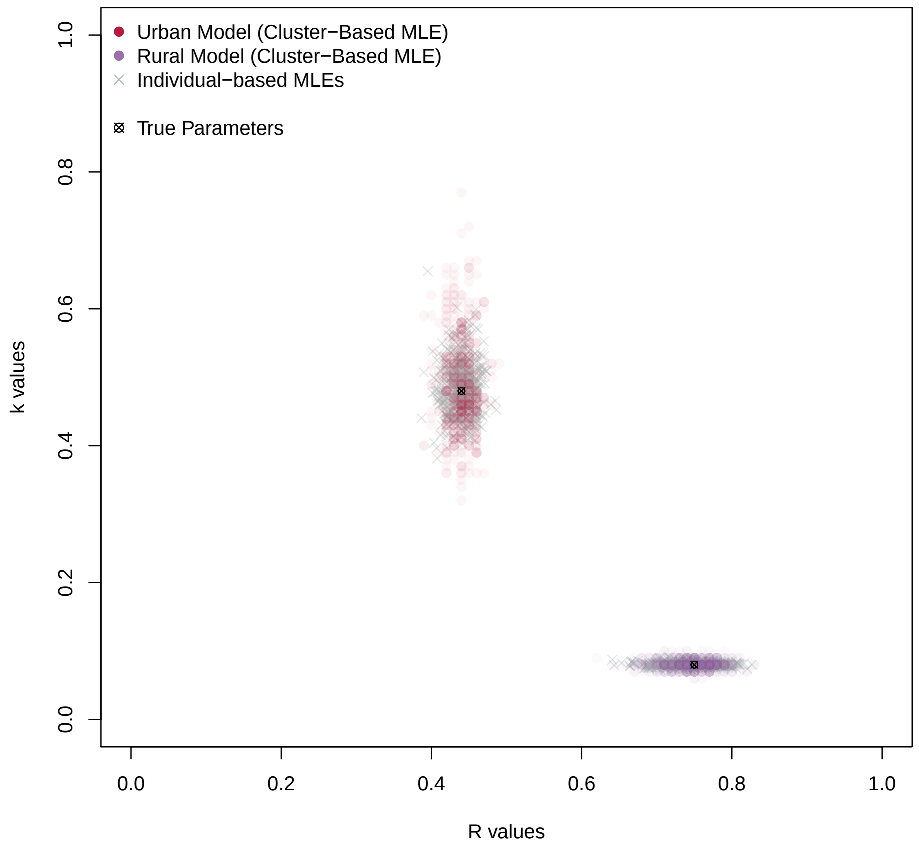

2.6 Supplemental Table S6: Impact of missing cases on inference of k.

Probability of case ascertainment by passive and active surveillance. Numbers in the center of each tile represent the median $\hat{k}$ value of 500 simulated surveillance systems with 2000 chains for each combination.

(a) Urban model ($R = 0.44, k = 0.48$)

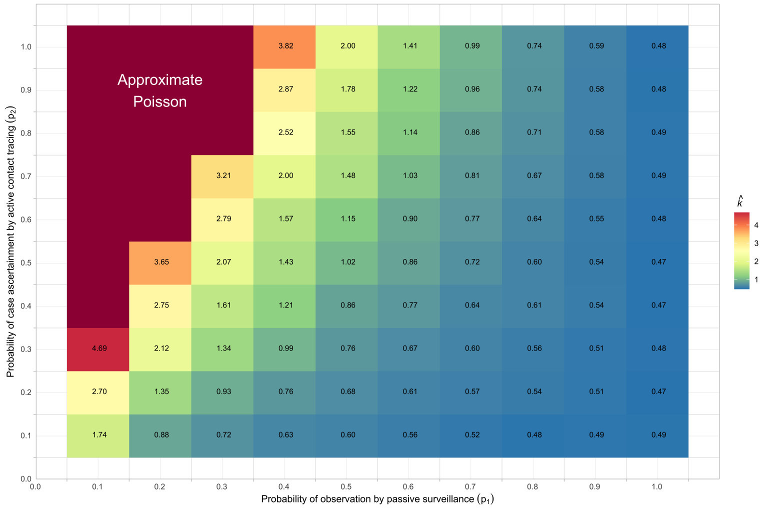

(b) Rural model ($R = 0.75, k = 0.08$)

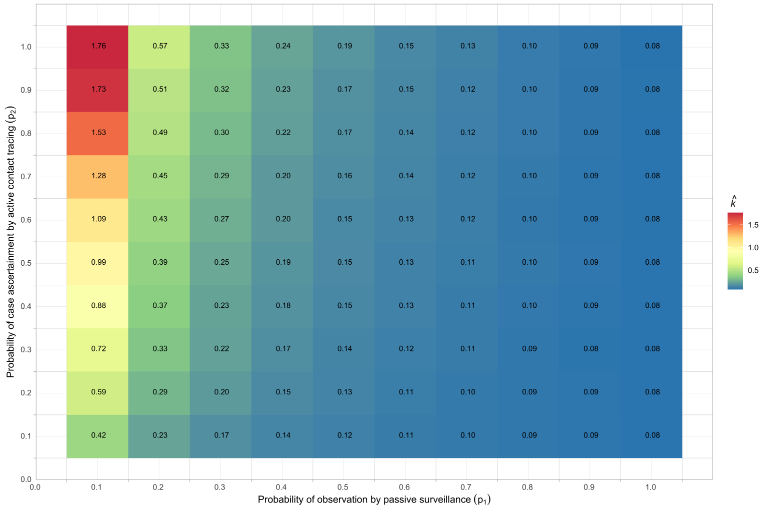

2.7 Supplemental Figure S7: Assessing the conditional approach to sub-clustering.

We simulated MIRU-VNTR clusters with transmission sub-clustering in 100 surveillance systems, each with 2000 transmission chains, for each model. Estimates of k (circles) were highly sensitive and overestimated k when sub-clustering was unaccounted for in the likelihood (red circles). Conditioning on the likelihood sufficiently corrected for transmission sub-clustering (green circles). (A) Urban model (true $R = 0.44, k = 0.48$); (B) Rural model (true $R = 0.75, k = 0.08$)

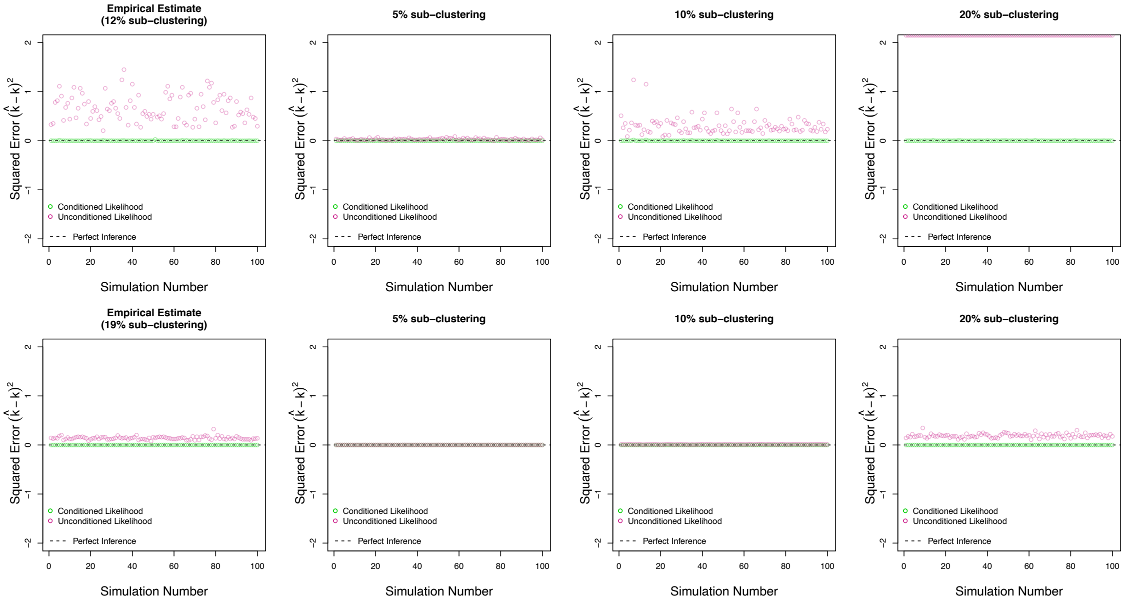

2.8 Supplemental Figure S8: Assessing the impact of censorship on parameter inference.

We simulated 500 perfect and imperfect surveillance systems, each with 2000 transmission chains, for each model. Imperfect surveillance assumed the proportion of censored clusters were consistent with empirical data for the respective populations (approximately 5 percent and 20 percent in the urban and rural populations, respectively). Censoring appears to bias inference of k downward.

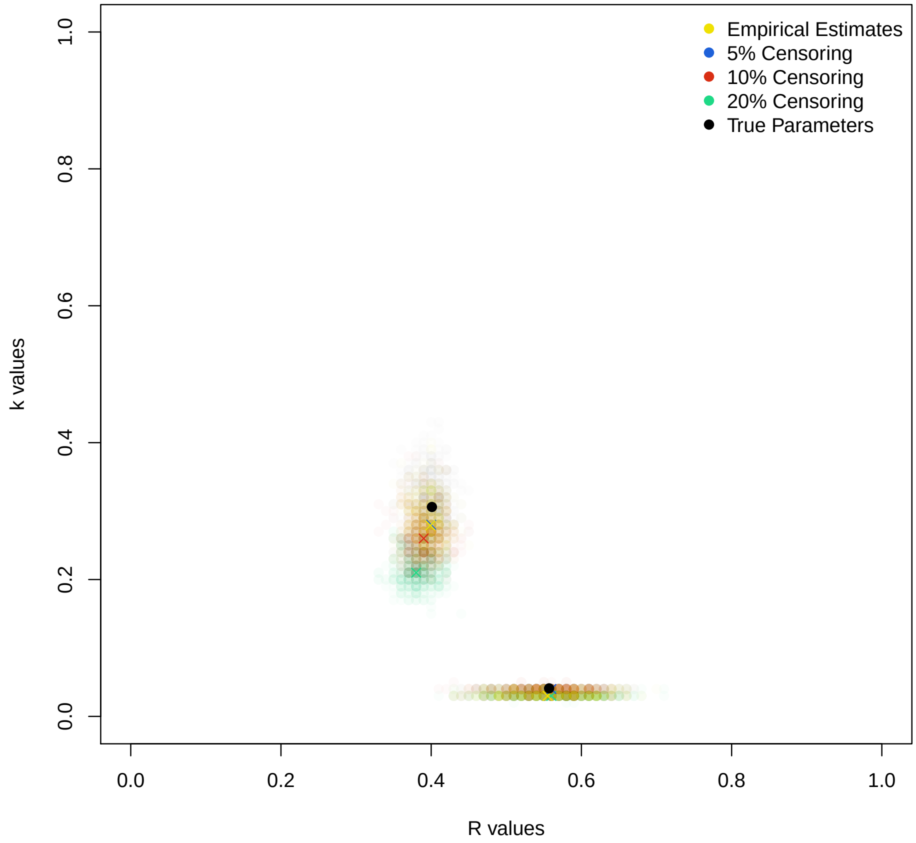

Supplement: Supplementary file 1 — Supplementary Information. [file 41598_2022_10488_MOESM1_ESM.pdf]
